# Supplementary material for: Post-Acute Sequelae of SARS-CoV-2 Infection (PASC) for Patients—3-Year Follow-Up of Patients with Chronic Kidney Disease
Source: Biomedicines. 2024 Jun 5;12(6):1259. doi: 10.3390/biomedicines12061259 (PMC11201278; doi:10.3390/biomedicines12061259)
Supplement: Supplementary file 1 [file biomedicines-12-01259-s001.zip › biomedicines-3022051-supplementary.pdf]

biomedicines

MDPI

Supplementary Materials

UMHAT SAINT ANNA

UNIVERSITY HOSPITAL

BULGARIA, SOFIA

Dimitar Mollov street, #1

Nephrology department

Patient Information:

- Name:
- Age:
- Date of COVID-19 Diagnosis:
- Current CKD Stage:
- Which in row is the current visit in the outpatient clinic:

COVID-19 Recovery and Long-Term Symptoms Questionnaire

1. **COVID-19 History:**
    - Date of COVID-19 diagnosis:
    - Duration of COVID-19 symptoms:
    - Hospitalization history (if any):
    - Did you require oxygen therapy or mechanical ventilation during your COVID-19 illness? (Yes/No):
2. **Current Symptoms:** Please mark the severity and frequency of each symptom experienced since recovering from COVID-19. Use the following scale:
    - Fatigue:
    - Shortness of breath:
    - Chest pain or tightness:
    - Cough:
    - Muscle or joint pain:

- Headaches:
- Difficulty concentrating or brain fog:
- Loss of taste or smell:
- Palpitations or irregular heartbeat:
- Gastrointestinal symptoms (e.g., nausea, diarrhea):
- Skin rashes or changes:
- Other (please specify):

3. **Kidney Health:**

- Have you noticed any changes in your kidney function since recovering from COVID-19? (e.g., changes in urine output, increased blood pressure, swelling in legs)

0	Not present
1	Mild
2	Moderate
3	Severe

- Are you experiencing any new or worsening symptoms related to your CKD since COVID-19 recovery? Please describe:

4. **Mental Health:**

- How would you rate your overall mental health post-COVID-19 recovery? (Good/Fair/Poor)
- Have you experienced increased anxiety, depression, or mood changes since recovering from COVID-19?

Date:

УНИВЕРСИТЕТСКА БОЛНИЦА "СВЕТА АННА",
БЪЛГАРИЯ, СОФИЯ
Улица "Димитър Моллов", № 1
Отделение по нефрология

Информация за пациента:

- Име:
- Възраст:
- Дата на поставяне на диагнозата COVID-19:
- Текущ стадий на ХБЗ:
- Който ред е текущото посещение в амбулаторията:

Въпросник за възстановяване и дългосрочни симптоми COVID-19

1. **COVID-19 История:**
    - Дата на диагностициране на COVID-19:
    - Продължителност на симптомите на COVID-19:
    - История на хоспитализациите (ако има такива):
    - Нуждаехте ли се от кислородна терапия или механична вентилация по време на заболяването си от COVID-19? (Да/Не):
2. **Настоящи симптоми:** Моля, отбележете тежестта и честотата на всеки симптом, който изпитвате след възстановяването от COVID-19. Използвайте следната скала:

0	Не присъства
1	Леко
2	Умерен
3	Тежък

- Умора:
- Задух:
- Болка или стягане в гърдите:
- Кашлица:
- Мускулна или ставна болка:
- Главоболие:
- Затруднена концентрация или мозъчна мъгла:
- Загуба на вкус или мирис:

- Палпитации или неравномерен сърдечен ритъм:
- Стомашно-чревни симптоми (напр. гадене, диария):
- Кожни обриви или промени:
- Друго (моля, посочете):

3. **Здраве на бъбреците:**

- Забелязали ли сте някакви промени в бъбречната си функция след възстановяването от COVID-19? (напр. промени в отделянето на урина, повишено кръвно налягане, подуване на краката)
- Имате ли някакви нови или влошаващи се симптоми, свързани с Вашата ХБН, след възстановяването на COVID-19? Моля, опишете:

4. **Психично здраве:**

- Как оценявате цялостното си психично здраве след възстановяването от COVID-19? (добро/добро/слабо)
- Имали ли сте повишена тревожност, депресия или промени в настроението след възстановяването от COVID-19?
